# Intra-thoracic fat volume is associated with myocardial infarction in patients with metabolic syndrome

**DOI:** 10.1186/1532-429X-15-77

**Published:** 2013-09-10

**Authors:** Umjeet S Jolly, Abraam Soliman, Charles McKenzie, Terry Peters, John Stirrat, Immaculate Nevis, Matthew Brymer, Tisha Joy, Maria Drangova, James A White

**Affiliations:** 1 Department of Medicine, Schulich School of Medicine and Dentistry, University of Western Ontario, London, Ontario, Canada; 2 Robarts Research Institute, University of Western Ontario, London, Ontario, Canada; 3 Lawson Health Research Institute, University of Western Ontario, London, Ontario, Canada; 4 Department of Medical Biophysics, University of Western Ontario, London, Ontario, Canada; 5 Biomedical Engineering, University of Western Ontario, London, Ontario, Canada; 6 Cardiovascular MRI Clinical Research (CMCR) Program, London Health Sciences Center, 339 Windermere Road, London, Ontario N6A 5A5, Canada

**Keywords:** Fat distribution, Adiposity, Metabolic syndrome, Myocardial infarction

## Abstract

**Background:**

Visceral adiposity is increased in those with Metabolic Syndrome (MetS) and atherosclerotic disease burden. In this study we evaluate for associations between intra-thoracic fat volume (ITFV) and myocardial infarction (MI) in patients with MetS.

**Methods:**

Ninety-four patients with MetS, MI or both were identified from a cardiovascular CMR clinical registry. MetS was defined in accordance to published guidelines; where-as MI was defined as the presence of subendocardial-based injury on late gadolinium enhancement imaging in a coronary vascular distribution. A healthy control group was also obtained from the same registry. Patients were selected into the following groups: MetS+/MI- (N = 32), MetS-/MI + (N = 30), MetS+/MI + (N = 32), MetS-/MI- (N = 16). ITFV quantification was performed using signal threshold analysis of sequential sagittal CMR datasets (HASTE) and indexed to body mass index.

**Results:**

The mean age of the population was 59.8 ± 12.5 years. MetS+ patients (N=64) demonstrated a significantly higher indexed ITFV compared to MetS- patients (p = 0.05). Patients in respective MetS-/MI-, MetS+/MI-, MetS-/MI+, and MetS+/MI + study groups demonstrated a progressive elevation in the indexed ITFV (22.3 ± 10.6, 28.6 ± 12.6, 30.6 ± 12.3, and 35.2 ± 11.4 ml/kg/m2, (p = 0.002)). Among MetS+ patients those with MI showed a significantly higher indexed ITFV compared to those without MI (p = 0.02).

**Conclusions:**

ITFV is elevated in patients with MetS and incrementally elevated among those with evidence of prior ischemic myocardial injury. Accordingly, the quantification of ITFV may be a valuable marker of myocardial infarction risk among patients with MetS and warrants further investigation.

## Background

Visceral adiposity is a recognized phenotypic feature of Metabolic Syndrome (MetS) and may be a quantifiable imaging marker of atherosclerotic disease risk [1-3]. The intra-thoracic fat volume (ITFV) is one such marker shown to be reproducible by thoracic computed tomography (CT) imaging, and appears to correlate with other validated cardiovascular risk prediction models [4,5] as well as serologic markers of vascular inflammation [6]. ITFV has been associated with the extent of coronary artery calcification [7], suggesting association with coronary artery disease (CAD) burden. This measure has also been shown to be independently predictive of future cardiovascular events among a broad referral population receiving cardiac CT for the evaluation of acute chest pain [8]. However, among patients with MetS, whether ITFV quantification may discriminate risk of ischemic myocardial injury remains unclear.

Within this cohort study we explore associations between ITFV and the presence of ischemic myocardial injury (ie: myocardial infarction) among patients meeting criteria for MetS. ITFV measures were compared to control populations of patients without MetS both with and without prior myocardial infarction. A novel, rapid cardiovascular magnetic resonance (CMR) based approach to the quantification of ITFV was employed and validated.

## Methods

### Patient population

Ninety-four patients with metabolic syndrome (MetS) and/or myocardial infarction (MI) undergoing Late Gadolinium Enhancement (LGE) were identified from a clinical CMR registry at the Cardiovascular MR Clinical Research (CMCR) program between March 2009 and March 2011. MetS was defined in accordance with published guidelines [9] as any three of the following five criteria: body mass index (BMI) ≥30 kg/m^2^, serum triglycerides ≥1.7 mmol/L, high density lipoprotein C (HDL-C) ≤1.0 mmol/L for men or ≤1.3 mmol/L for women, Hemoglobin A_1_C ≥6.5%, systolic blood pressure ≥130 mm Hg or diastolic blood pressure ≥85 mm Hg. Current pharmacologic therapy for hyperlipidemia, diabetes, or hypertension was considered equivalent to their respective criteria. The presence of MI was defined as the unequivocal presence of subendocardial-based (ie: ischemic) enhancement on LGE CMR, confirmed in two imaging planes. In addition, 16 normal subjects were identified to serve as a control population for ITFV measurement, each required to have no history of MetS or overt cardiovascular disease.

Based upon the established inclusion criteria 4 groups were available for analysis: MetS without MI (MetS+/MI-) [N = 32], MetS with MI (MetS+/MI+) [N = 32], No MetS with MI (MetS-/MI+) [N = 30], and No MetS and No MI (MetS-/MI-) [N = 16].

Patients with a Glomerular Filtration Rate (GFR) of ≤30 ml/min/1.73 m^2^ were excluded due to FDA recommendation against the administration of gadolinium-based contrast agents in this population [10]. Patients with standard contraindications to CMR were similarly excluded. Ethics approval was obtained for this study from the University of Western Ontario Research Ethics Board.

### Cardiovascular MR protocol

**Figure 1:**
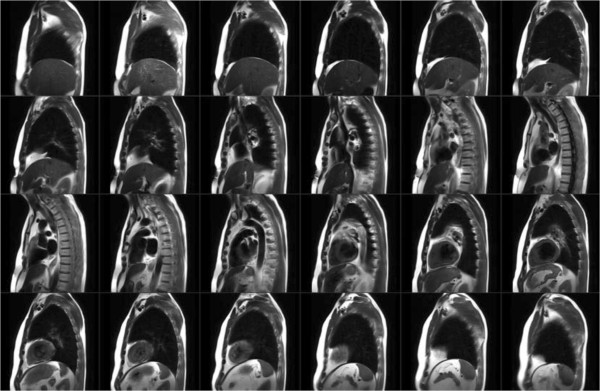
**Example images using free-breathing magnetic resonance imaging in sagittal plane employing the HASTE pulse sequence.** Adipose tissue demonstrates high signal relative to all other tissues.

CMR was performed using 3 T MRI scanners (TIM Trio or Verio, Siemens, Erlangen, Germany). Images were obtained using a 32-channel phased-array radiofrequency coil with ECG-gating. All patients had thoracic imaging performed in the sagittal orientation inclusive of both humeral heads using a Half-Fourier Acquisition Single-shot Turbo-spin Echo (HASTE) pulse sequence during shallow breathing (slice thickness 8 mm, gap 2 mm, matrix 256 x 155, FOV 400–450 x 350-400 mm, TE 50 ms, TR 600 ms, iPAT 2). The typical imaging time required for this survey was 25–30 seconds (1 image slice per cardiac cycle), depending upon heart rate (See Figure 1). Cardiac function was assessed in the short-axis orientation at 10-mm intervals from the atrioventricular annulus to apex and in the 2, 3 and 4-chamber views using a standard SSFP-based pulse sequence (slice thickness 6-mm, gap 4-mm, TE 1.3 ms, flip angle 10 degrees, matrix 256x205, iPAT 2, temporal resolution 28-38 ms). Ten to fifteen minutes following the intravenous administration of Gadolinium contrast (0.1 to 0.2 mmol/kg, Gadovist®, Bayer, Inc), LGE imaging was performed using a standard segmented inversion-recovery gradient echo pulse sequence in imaging planes identical to cine images. Typical imaging parameters were: slice thickness = 6 mm; gap = 4 mm; TR = 800 ms; TE = 3.9 ms; flip angle = 20 degrees; matrix 256 x 205; trigger pulse = 2; segments 13–21; iPAT = 2. The inversion time was optimized to null normal myocardium, as previously described [11].

### CMR image analysis: cine and delayed enhancement imaging

All cine and LGE-CMR datasets were blindly analyzed by an experienced CMR interpreter as part of a standardized, core-laboratory protocol. All quantitative analysis was performed using the same commercially available analysis software (CMR42, Circle Cardiovascular Inc, Calgary). The LV end-systolic volume (ESV), LV end-diastolic volumes (EDV) and LV mass were obtained using semi-automated contour tracing of sequential short axis cine image datasets. LGE imaging was visually scored for the presence of any abnormal enhancement and its transmural distribution. Only patients with unequivocal subendocardial contrast enhancement in two or more contiguous / orthogonal image slices not associated with artifact were labeled as having MI. Signal quantification of LGE images was incrementally performed using a Signal Threshold Versus Reference Myocardium (STRM) technique to measure infarct mass, as previously described [12]. This technique was performed by manual tracing of the endocardial and epicardial contours with the tracing of the largest contiguous region of normal reference myocardium. Total scar volume was determined using a signal threshold of ≥5SD above the mean signal of normal reference myocardium. Total infarct mass was expressed as a percentage of the total LV mass.

### CMR image analysis: thoracic fat quantification

**Figure 2:**
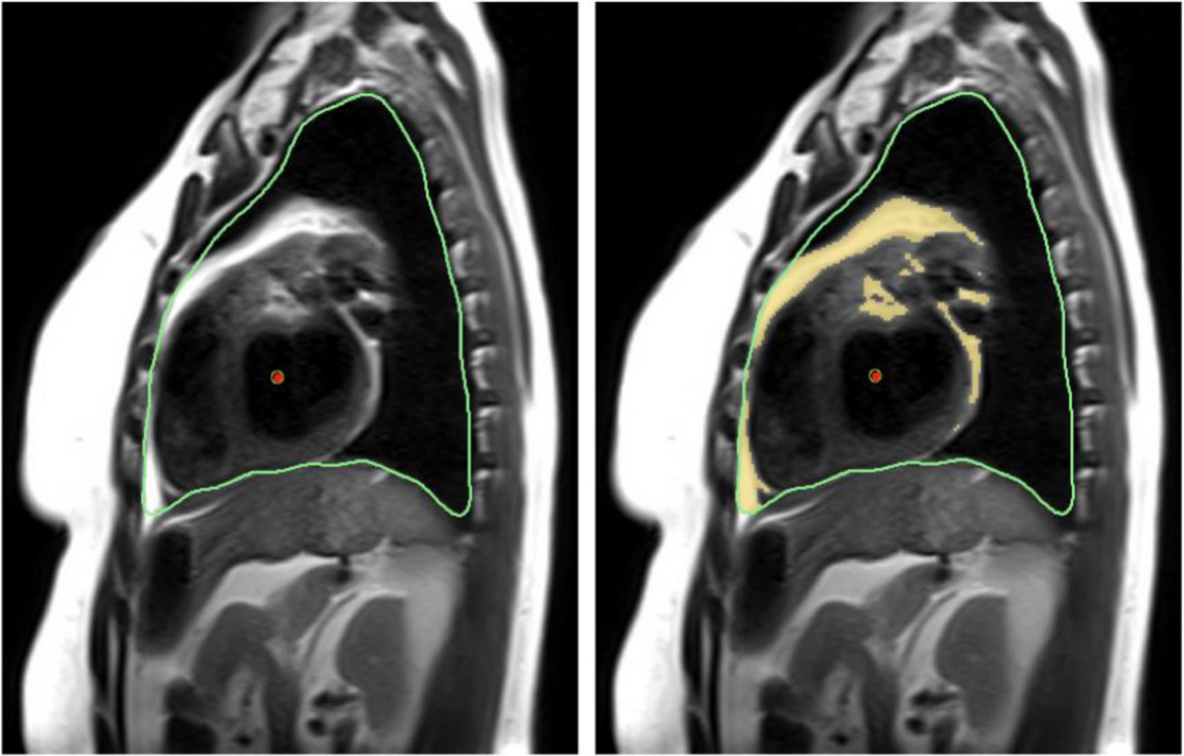
**Example of signal threshold based analysis technique from a mid thoracic sagittal HASTE image.** Green line = Outer limits (parietal thoracic border), Red line = Inner limits (cardiac chamber shown), Blue line = reference tissue (paravertebral skeletal muscle). Yellow = segmented fat signal.

To determine a standardized signal threshold for fat quantification on HASTE imaging we performed a visual calibration study, referencing CT-based fat segmentation performed using previously validated techniques [7,13,14]. This was performed in 10 study patients who had also received cardiac CT imaging within one month of their CMR study. In these patients their CMR and CT datasets were simultaneously displayed, the latter re-formatted using a 3D multi-planar reconstruction (MPR) to provide anatomically matched slice orientations of three randomly selected MR imaging planes (total of 30 slices evaluated) (OsirX, Version 4.0). Manual contour tracing of the intra-thoracic borders was performed for all selected images, the anatomic limits defined as the level of the clavicle superiorly, the diaphragm inferiorly, the sternum anteriorly, and the vertebral bodies / ribs, posteriorly. An example of this is shown in Figure 2. A validated signal threshold fat signal range (−190 to −30 Hounsfield units [7,13,14]) was applied to highlight adipose tissue on the CT image. Five randomly selected regions of paravertebral muscle were selected on the corresponding CMR dataset to serve as a signal reference, the mean signal and SD of these regions determined using a 5–10 mm region of interest. This was followed by manual adjustment of the signal threshold on the CMR image, described by SD above this mean value, until a visual match of intra-thoracic fat segmentation was obtained between the two images. Using this process a signal threshold of ≥10 SD above the mean signal of paravertebral muscle was consistently found to be most effective in identifying intra-thoracic adipose tissue on HASTE images compared to CT-based segmentation.

CMR-based quantification of the ITFV was blindly performed on sequential sagittal HASTE images using manual contour tracing of the intra-thoracic border, as described above, followed by applying the signal threshold of ≥10 SD above the mean signal of paravertebral muscle. The total ITFV was derived as the sum of fat signal area from all slices multiplied by the total slice thickness. ITFV was indexed to the patients’ body mass index (BMI). Total analysis time for measurement of ITFV from HASTE imaging ranged from 8 to 14 minutes.

Intra-observer and inter-observer variability of ITFV quantification was evaluated by the repeated measurement of 10 randomly selected study patients by two interpreters. This was performed in random order on two separate occasions.

### Statistical analysis

Continuous variables are presented as mean ± SD or median (range) depending on distribution. Parameters with normal distribution were tested using independent-sample *t* test (2-tailed distribution) for two group comparison whereas means of more than two groups were compared using Analysis of Variance (ANOVA). For non-normally distributed continuous data (reported as medians and range), the Mann–Whitney U or Kruskal Wallis test was used. Categorical data are summarized as frequencies and percentages between groups using the chi-square test or the Fisher exact test as appropriate.

Multivariable linear regression analysis was used to determine significant predictors of total scar volume. Reproducibility of ITFV analysis was assessed by linear regression analysis, and the Pearson correlation coefficient (r) is reported for inter- and intra- observer variability of both measures. Bland and Altman analysis was performed to evaluate for systematic bias.

All statistical tests were two-tailed and p <0.05 was regarded as significant. S-Plus (version 8.0, Insightful Software, Seattle, WA) was used to perform the statistical analyses.

## Results

### Baseline patient characteristics

Baseline clinical characteristics are shown in Table 1. The mean age of the study population was 59.8 ± 12.5 years with 78% being male and 71% being Caucasian. Patients with MetS were more likely to be receiving oral hypoglycemic therapy, lipid lowering therapy and anti-hypertensive therapy. Higher triglyceride levels were seen in those with MetS while LDL values were modest due to concurrent pharmacologic therapy.

**Table 1:** Baseline clinical characteristics according to patient cohort (Total N = 110)

**Characteristic**	**MetS-MI- (n = 16)**	**MetS + MI- (n = 32)**	**MetS-MI + (n = 30)**	**MetS + MI + (n = 32)**
Age (years)	42.3 ± 16.9	62.0 ± 10.2	60.1 ± 11.4	58.7 ± 14.8
Female sex (%)	7 (44%)	7 (23%)	4 (14%)	3 (9%)
Caucasian (%)	10 (62%)	25 (81%)	23 (79%)	26 (79%)
BMI (kg/m^2^)	22.9 (21.7 – 24.0)	32.5 (30.3 – 34.8)	26.0 (24.5 – 27.5)	30.4 (28.7 – 32.0)
Waist circumference (cm)	88.7 ± 25	119.9 ± 9	106.4 ± 14.3	116.0 ± 11
eGFR (ml/minute)	94 ± 18	81 ± 23	78 ± 20	76 ± 21
Comorbidities (%)				
Hypertension	0 (0%)	24 (77%)	13 (45%)	23 (70%)
Diabetes	0 (0%)	14 (45%)	2 (7%)	14 (42%)
Type I	0	2	0	0
Type II	0	12	2	14
Hyperlipidemia	0 (0%)	28 (90%)	16 (55%)	24 (73%)
Smoking	2 (12%)	13 (42%)	11 (38%)	15 (46%)
Prior PCI or CABG	0 (0%)	10 (32%)	12 (41%)	19 (58%)
Blood profile				
HgbA1c	NA	0.08 (0.06 – 0.09)	0.06 (0.05 – 0.07)	0.07 (0.05 - 0.07)
Total cholesterol	4.9 (3.8 – 6.1)	3.8 (3.4 – 4.3)	4.2 (3.6 – 4.7)	4.0 (3.5 – 4.5)
Triglyceride	1.2 (0.8 – 1.6)	2.0 (1.6 – 2.5)	1.3 (0.9 – 1.6)	1.9 (1.3 – 2.4)
HDL	1.3 (0.8 – 1.7)	0.9 (0.8 – 1.0)	1.2 (1.0 – 1.3)	0.9 (0.8 – 1.0)
LDL	3.1 (2.2 – 4.1)	2.1 (1.6 – 2.5)	2.5 (1.9 – 3.0)	2.3 (1.8 - 2.7)
Total cholesterol/HDL ratio	4.1 (2.9 – 5.3)	4.3 (3.9 – 4.8)	3.9 (3.1 – 4.7)	4.7 (3.8 – 5.6)
Current medications				
Aspirin (ASA)	4 (25%)	15 (46.9%)	21 (70.0%)	22 (68%)
ACE inhibitor	2 (12.5%)	10 (31.3%)	21 (70.0%)	21 (65.7%)
ARB	0 (0%)	9 (28.1%)	3 (10%)	8 (25%)
Beta blocker	5 (31.3%)	26 (81.3%)	24 (80.0%)	25 (78.1%)
Diuretic	1 (6.3%)	11 (34.4%)	7 (23.3%)	17 (53.1%)
Fibrate	0 (0%)	1 (3.1%)	1 (3.3%)	2 (6.3%)
Insulin	0 (0%)	1 (3.1%)	0 (0%)	2 (6.3%)
Oral Hypoglycemic	0 (0%)	11 (34.4%)	2 (6.7%)	8 (25%)
Statin	2 (12.5%)	24 (75%)	16 (53.3%)	19 (59.4%)
Plavix	0 (0%)	4 (12.5%)	6 (20.0%)	7 (21.9%)

Results are presented as a mean with 95% confidence interval for continuous data or number with proportion for categorical data. *MetS* Metabolic Syndrome, *MI* Myocardial Infarction, *BMI* Body Mass Index, *PCI* Percutaneous Coronary Intervention, *CABG* Coronary Artery Bypass Grafting, *Hgb* Hemoglobin, *HDL* High density lipoprotein, *LDL* Low density lipoprotein, *ASA* Aspirin, *ACE* Angiotensin converting enzyme.

Of the 62 patients demonstrating LGE-CMR evidence of prior MI 10 (16%) were clinically silent with no history of a clinical event. The mean time interval between the most recent clinically recognized MI and CMR was 0.9 ± 2.3 years.

### Cardiovascular MR findings

#### Cine and delayed enhancement imaging

The results of cine and LGE-MRI analysis are summarized in Table 2. Significant differences in the LVEDV, LVEF and LV mass were identified between those with prior MI (MetS+/MI + and MetS-/MI+) and those without prior MI (p < 0.01 for all comparisons).

**Table 2:** Baseline CMR characteristics according to patient cohort (Total N = 110)

**Characteristic**	**MetS-MI- (n = 16)**	**MetS + MI- (n = 32)**	**MetS-MI + (n = 30)**	**MetS + MI + (n = 32)**
LV EDV (indexed to BSA)	78.9 (59.4–98.4)	80.2 (66.8–93.6)	99.8 (87.3–112.3)*	113.0 (98.3–127.6)*
LV EF (%)	63 (54 – 72)	58 (50 – 66)	40 (33 – 47)*	34 (29 – 40)*
LV mass (indexed to BSA)	71.2 (56.6-85.9)	74.1 (65.6-82.7)	82.6 (75.2-89.9) *	90.2 (81.8-98.6) *
Injury pattern on LGE CMR	0	0	30 (100%)	32 (100%)
−1 vascular territory	NA	NA	24 (80%)	18 (56.3%)
- ≥ 2 vascular territories	NA	NA	6 (20%)	14 (43.8%)
Total scar (% LV Mass)	2.1 ± 2.1	3.6 ± 7.5	21.1 ± 14.7*	21.0 ± 15.4*
Non-indexed ITFV (ml)	191 ± 125	506 ± 269*	500 ± 293*	668 ± 292*
Indexed ITFV (ml/kg/m^2^)	22.3 ± 10.6	28.6 ± 12.6*	30.6 ± 12.3*	35.2 ± 11.4*

Results are presented as a mean with 95% confidence interval for continuous data or number with proportion for categorical data. *Indicates p < 0.05 versus control population. *LV* Left Ventricle, *BSA* Body Surface Area, *EDV* End Diastolic Volume, *EF* Ejection Fraction, *DE* Delayed Enhancement, *ITFV* Intra-thoracic Fat Volume.

**Figure 3:**
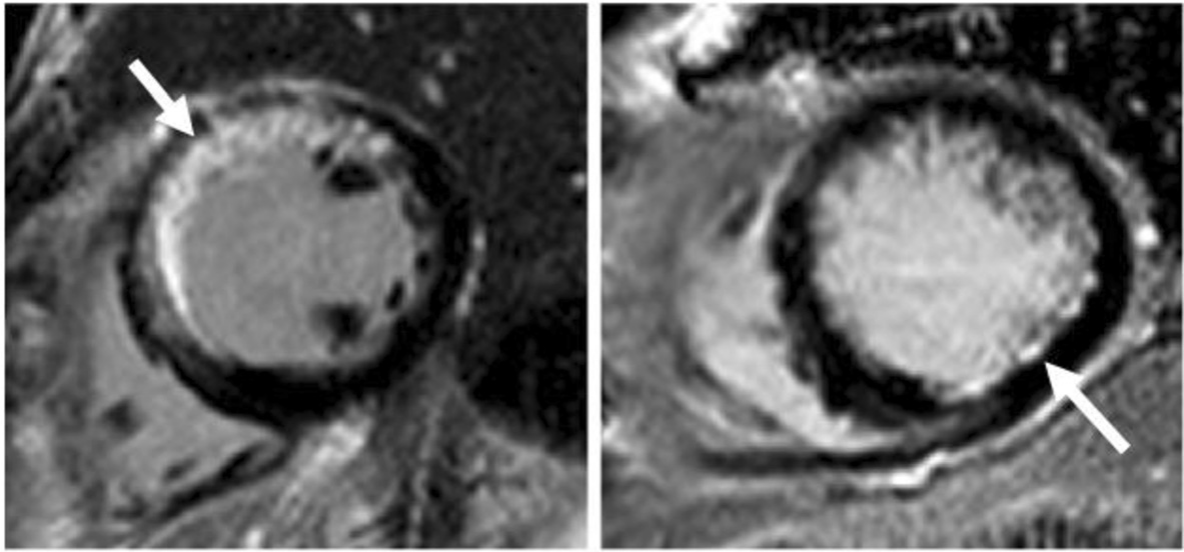
**Examples of myocardial infarctions identified by Late Gadolinium Enhancement imaging in patients with Metabolic Syndrome.** Left: Patient with a large, clinically recognized anteroseptal myocardial infarction. Right: Patient with a small, clinically silent subendocardial infarction in the posterolateral wall.

The extent of myocardial infarction ranged from large, multi-territory transmural injury to small, discrete subendocardial injury, as shown in Figure 3. This injury was seen in a single coronary artery territory in 42 patients (68%) and ≥2 territories in 20 patients (32%). Using signal threshold-based quantification of LGE no differences in total infarct size was identified between those without MetS versus those with MetS (20.6 ± 14.2% versus 19.8 ± 13.5%, respectively, p = 0.97).

#### Intra-thoracic fat volume (ITFV) quantification

**Figure 4:**
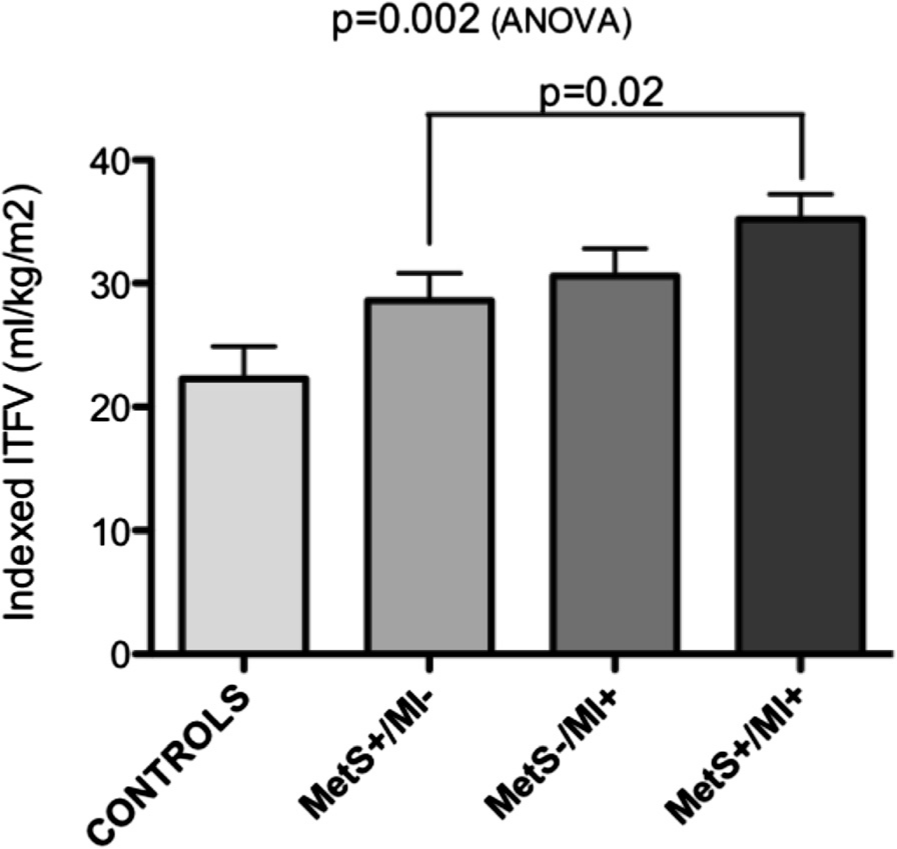
**Intra-thoracic Fat Volume (ITFV), indexed to body mass index, for Control patients and for the 3 disease patient cohorts.** MetS = Metabolic Syndrome. MI = Myocardial Infarction (as determined by LGE CMR).

The results of ITFV quantification are summarized in Table 2. The indexed ITFV was significantly different among the four patient cohorts (p = 0.002, ANOVA) (Figure 4). Incremental elevation in the ITFV was identified for the following cohorts; MetS+/MI–, MetS-/MI+, and MetS+/MI+, respectively, each demonstrating statistically significant elevations when compared to control subjects (p = 0.08, p = 0.02, p = 0.0005, respectively). Among patients with MetS, the indexed ITFV was significantly higher among those with LGE-MRI evidence of MI versus those without MI (35.2 ± 11.4 ml/kg/m^2^ vs 28.6 ± 12.6 ml/kg/m^2^, p = 0.02).

Multivariable linear regression analysis was performed among the entire population to explore for independent associations with total infarct volume. The following variables were entered into this model; indexed ITFV, age, sex, race, BMI, diabetes and GFR. This revealed indexed ITFV to be the strongest independent predictor of infarct volume following adjustment for all other variables (Beta 0.8, p = 0.01). A significant association (ie: co-linearity) was present between indexed ITFV and both waist circumference and hypertension (p = 0.005), preventing their inclusion in the same model. However, replacement of indexed ITFV with either variable resulted in a lower strength of association, suggesting indexed ITFV to be more robustly associated with infarct burden.

#### Intra and inter-observer variability

**Figure 5:**
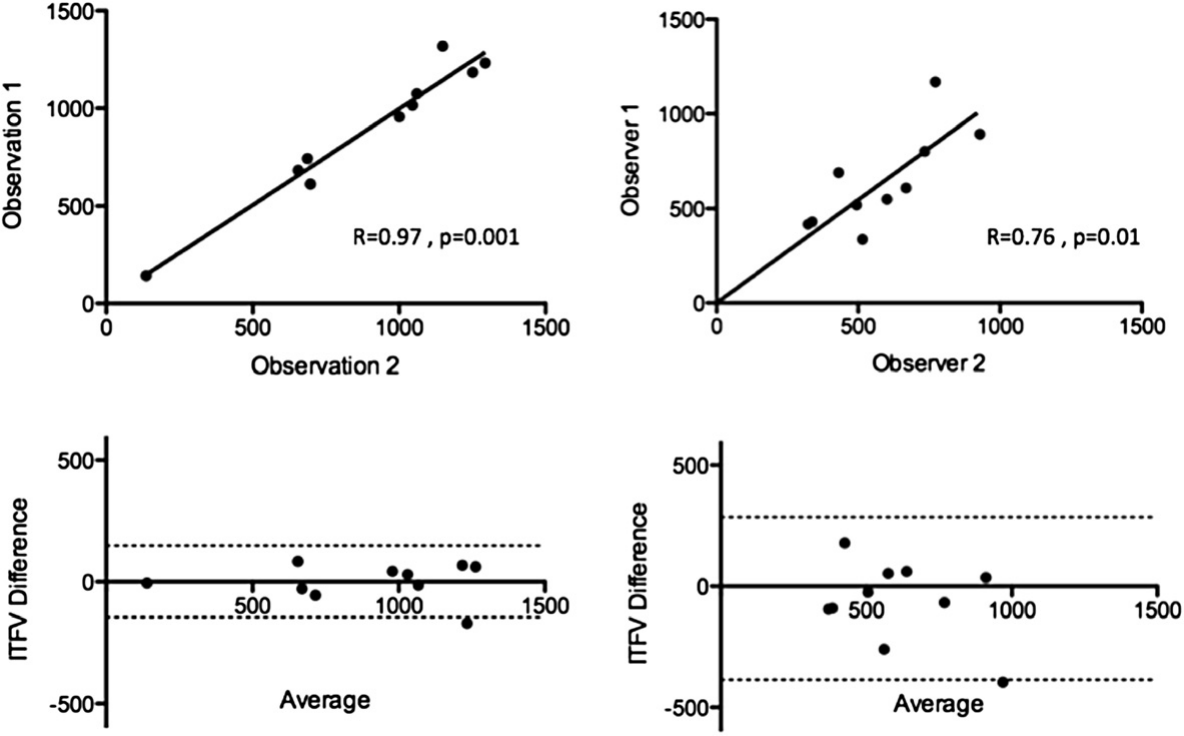
Results of Intra-observer and Inter-observer reproducibility testing for ITFV measurement from HASTE CMR, shown using both linear regression analysis (upper) and Bland-Altman analysis (lower).

Intra-observer reproducibility for ITFV measurements was excellent with a Pearson correlation coefficient of 0.97, while the inter-observer data showed a coefficient of 0.76 (Figure 5). Bland and Altman analyses demonstrated no significant bias for either intra-observer or inter-observer testing [1.2 (95% CI −146.3 to 148.7), and −60.5 (95% CI −386.7 to 265.7), respectively].

## Discussion

This study is the first to identify an association between thoracic visceral adiposity and the occurrence of myocardial infarction. Our results indicate that, among a sampled cohort of patients undergoing CMR imaging, ITFV is both associated with MetS and is incrementally elevated among those with objective evidence of myocardial infarction.

An expanding body of evidence supports that MetS patients experience an alteration in adipose tissue distribution to the visceral surfaces of abdominal [15] and mediastinal organs [16]. These two visceral fat repositories are recognized to have common embryologic origins [2] and share similar metabolic profiles that contribute to increased production of inflammatory cytokines, such as; interleukin-6, interleukin-8, and TNF-α. [17]. These mediators may exert both a local cytotoxic [18] and systemic inflammatory [17] influence upon vascular endothelium, potentially initiating or propagating the development of atherosclerosis. This theory is supported by a study showing a correlation between CT-based ITFV measures and serum levels of hsCRP [6], a validated systemic inflammatory marker of CAD risk [17,19,20].

Evidence for an increased prevalence of atherosclerotic disease in patients with higher intra-thoracic fat volumes has been reported in studies employing thoracic CT imaging [6,7]. These studies, similarly executed from retrospective cohort data, evaluated patients receiving clinical cardiac CT and showed an association between ITFV and coronary artery calcification. The first study, reported by Dey *et al*. [6], identified that ITFV was significantly higher in those with versus without MetS (p < 0.0001) and showed a strong correlation with the coronary calcium score (r = 0.79, p <0.0001). In a second study, by Yun *et al.*[7], patients within the highest quartile of ITFV had a four-fold higher prevalence of coronary artery calcification. In combination, these studies provided compelling evidence that thoracic visceral adiposity is associated with a higher prevalence of coronary atherosclerosis. Interestingly, a recent study by Brinkley, *et al.*[21] failed to show pericardial fat volume, a component of ITFV, to be independently associated with hyperemic myocardial blood flow or myocardial perfusion reserve. However, this study was performed in asymptomatic low risk individuals with a low prevalence of diabetes.

The validation of thoracic fat volume measurements for the prediction of CAD-related events in patients with MetS is clinically attractive. Incremental discrimination of high-risk individuals more likely to benefit from early and aggressive pharmacologic intervention or ischemia surveillance is of importance. Farouzandeh *et al.* recently showed ITFV by cardiac CT to be independently associated with major adverse cardiovascular events among a broad population of patients referred for acute chest pain [8]. Our study provides evidence that ITFV is elevated in patients with MetS, but incrementally elevated among those with MetS experiencing ischemic myocardial events. This suggests that this imaging biomarker may of value for the discrimination and monitoring of cardiovascular risk among patients with MetS. Accordingly, we feel there is strong impetus and justification for related prognostic studies to be engaged in this patient population.

While echocardiography is highly accessible, and provides reproducible linear measures of pericardial fat thickness [20], these results appear to be less well correlated with true volumetric measures of visceral adiposity or systemic metabolic abnormalities [22]. While both routine CMR and CT protocols offer tomographic imaging that can provide appropriate datasets for intra-thoracic fat quantification, the lack of ionizing radiation with CMR techniques offers a particular advantage. Routine SSFP-based cine imaging can provide for simple linear measurements of pericardial fat thickness, and has been shown to be predictive of future atrial fibrillation [23]. However, such measurements are inherently dependent upon the architecture of pericardial fat relative to prescribed imaging planes, and may not accurately represent total visceral fat. Fat-water separation techniques are of increasing importance for accurate fat quantification and offer a potentially robust approach to volumetric assessments [24]. While currently still in a development phase, these techniques are of particular interest for future clinical research. The advantage of our currently described technique is that it is simple, immediately available, and reliably performed during shallow, free breathing in under one minute. Further, the images can be prescribed as standard “localizers” for the initiation of routine cardiac imaging protocols.

### Study limitations

As a retrospective cohort study, performed within a single-center CMR clinical registry, these findings require confirmation within a larger multi-center setting.

The CMR protocol incorporated the use of gadolinium-based contrast for the performance of LGE imaging. Accordingly, patients with significant renal insufficiency (GFR <30 ml/min/1.73 m^2^) were not included due to FDA warnings of its use in such patients. Extrapolation of study findings to such patients may not be appropriate.

The available healthy control population (MetS-/MI-) from the employed registry was modest in size due to the registry’s mandate to recruit patients with known or suspected cardiovascular disease. Accordingly, case matching of age and sex variables was not feasible and potentially introduces bias for comparisons among this group. However, significant differences in ITFV were identified between disease cohorts (ie: MetS+/MI- versus MetS+/MI+), supporting that ITFV is indeed associated with the occurrence of MI among those with MetS.

Finally, the small number of female patients, most notably in the MetS+/MI- and MetS-/MI- groups, presents a limitation for generalizability of our current findings. Therefore, we recommend validation of our findings within a larger population with more balanced gender representation.

## Conclusions

Intra-thoracic visceral fat, as represented by an indexed ITFV, is elevated among patients with MetS, is incrementally elevated among those suffering from myocardial infarction and appears to be an independent predictor of total infarct burden. These findings justify the performance of larger, prospective cohort studies evaluating the utility of ITFV measurement for the prediction of future cardiovascular events among patients with MetS.

### Consent

Written informed consent was obtained from the patient for the publication of this report and any accompanying images.

## Abbreviations

MetS: Metabolic syndrome; ITFV: Intra-thoracic fat volume; LGE: Late gadolinium enhancement; MI: Myocardial infarction; LV: Left ventricle; EF: Ejection fraction; CMR: Cardiovascular magnetic resonance; EDV: End diastolic volume; ESV: End systolic volume; STRM: Signal threshold versus reference myocardium.

## Competing interest

The authors declare that they have no competing interests.

## Authors’ contributions

JAW is the senior and corresponding author and was involved with study design, conception, data interpretation, manuscript revision and final approval. UJ and AS performed data analysis and manuscript preparation. JS and M.B. assisted in image analysis. TJ provided clinical expertise for study design, manuscript construction and revision. CM, TP and MD provided guidance for image analysis techniques and revision of the manuscript. All authors have read and approved the final manuscript.
